# Detection of Cobalamin and In Vitro Cell Imaging Based on Nitrogen-Doped Yellow Fluorescent Carbon Dots with Nano Architectonics

**DOI:** 10.3390/ma15249057

**Published:** 2022-12-18

**Authors:** Guang Yu, Yujia Cheng, Zhuohua Duan

**Affiliations:** Mechanical and Electrical Engineering Institute, Zhongshan Institute, University of Electronic Science and Technology of China, Zhongshan 528400, China

**Keywords:** fluorescent nanomaterials, carbon dots, Y-CDs, cell imaging probe

## Abstract

As novel fluorescent nanomaterials, carbon dots have attracted increasing research attention because of their simple synthesis, robust fluorescence, low toxicity, and easy functionalisation. Previous research was focused on preparing carbon dots from biomass and chemical materials; however, most of these carbon dots exhibited blue fluorescence. Moreover, the fluorescence quantum yield was generally low, significantly limiting their application in biological imaging. To broaden the application scope of carbon dots, this study prepared long-wavelength emitting nano-carbon dots that exhibited increased quantum yield. Novel N-doped yellow fluorescent nano-carbon dots (Y-CDs) were synthesised via a hydrothermal method using L-tartaric acid and urea as the precursors. The Y-CDs had a high quantum yield (15.9%) and demonstrated photostability at various pHs, temperatures, and ionic strengths. The Y-CDs could detect cobalamin effectively and selectively, showing a linear relationship between fluorescence intensity and cobalamin concentration. The related coefficient was 0.997, and the detection limit was 2.101 μmol/L. In addition, the Y-CDs were successfully used as an imaging probe for MDA-MB-231 cells. Therefore, the Y-CDs developed in this study can be used for cobalamin detection and cell imaging.

## 1. Introduction

Carbon dots (CDs) are a type of fluorescent nanomaterial that have a size of <10 nm. CDs were first synthesised in 2004. In that year, Professor Xu conducted cataphoresis to purify single-walled carbon nanotube fragments. In the process, the nanoparticles with fluorescence characteristics were separated. In 2006, Professor Sun used graphite and black charcoal as a raw material for the synthesis of fluorescent nanoparticles [1,2,3,4]. These nanoparticles exhibited improved fluorescence. It was the first time CDs were nominated officially. Compared with organic dyestuff and quantum dots, CDs possess good luminescence properties, have an easy synthesis route, and exhibit low consumption and high biocompatibility and fluorescence without bleaching [5,6,7]. Therefore, the research on CDs has attracted widescale attention. At present, there are two main methods for CD synthesis, namely, the top-to-bottom approach based on the bulk material and the bottom-to-top approach based on the small molecule. During early research, graphite and carbon fibre were mainly used as raw materials. The preparation methods included arc discharge, laser corrosion, or electrochemistry. The CDs synthesised by the abovementioned two methods mainly emit blue short-wavelength light, and their quantum efficiency is low [8,9]. Upon further research, CD synthesis was conducted using raw materials such as biomass, e.g., strawberries, lime juice, and silkworm chrysalises. Moreover, carbon-based molecules such as citric acid, urea, and ethylenediamine (EDA) were used [10,11]. The synthesis methods involved microwave and ultrasonic irradiation and the hydrothermal method because they are rapid and convenient. The current research focuses on the synthesis and broad-scale application of long-wavelength fluorescent CDs. Previous [12,13] experiments suggest that the most effective CD-synthesis method is nitrogen doping. This efficiency is due to the similarity in the radii of nitrogen and carbon atoms. The electronegativity of nitrogen is higher than that of carbon [14]. Doped nitrogen atoms can provide an extra lone pair of electrons to carbon atoms. Thus, upon nitrogen doping, the internal structure of carbon atoms changes, which improves the luminescent properties of N-doped CDs. Professor Qian used 1,2-EDA, 1,3-propylenediamine (PDA), 1,4-tetramethylenediamine, and ethylene glycol as the dopants. Their experimental results revealed that the different nitrogen contents of the as-prepared CDs induce different effects on the luminescent properties of the CDs [15,16]. The quantum yield (QY) of N-doped CDs was found to be 20.4–36.3%, which is considerably higher than that of undoped CDs. Professor Reckmeier used the hydrothermal method to prepare carbon quantum dots (CQDs) through nitrogen doping. The fluorescence quantum efficiency of the CQDs was found to be 80%.

Unlike traditional fluorescent materials, CDs possess good biocompatibility because of their low toxicity. Thus, the side effect of CD entry into the human body is mitigated [17]. Moreover, the water solubility of most CDs is excellent; thus, sample preparation does not involve the use of complex pre-treatment methods. Finally, the fluorescence signal of CDs is stable and is less likely to be affected by the complex biological environment in the body [18,19]. Because of these advantages, CDs can be applied in the field of biological imaging. Professor Gong used amylaceum, EDA, and strong phosphoric acid (SPA) to prepare cavity CDs through phosphorus–nitrogen co-doping. These CDs were found to be applicable as a nanocarrier of doxorubicin anticancer drugs. The co-doped CDs also inhibited tumour growth, as determined by fluorescence imaging. Moreover, Professor Chen synthesised a series of hydrophobically modified CDs via the ring opening reaction. These CDs exhibited less cytotoxicity and good serum tolerance, and allowed [20] dual-channel imaging. They were also found to be effective for monitoring transitive DNA in cells. A cellular uptake experiment confirmed that such modified CDs exhibit good serum tolerance and a strong structure–activity relationship. Furthermore, Professor Yang conducted microwave-assisted carbonation for the synthesis of fluorine-doped CDs, which emitted red fluorescence. They also proposed a mechanism underlying the red fluorescence emission. Moreover [21], these CDs are used as an optical nanoprobe for biological imaging in the body. According to experimental results, the fluorine-doped CDs have promising applications in biological imaging or diagnosis of tumours. In summary, the application scope of CDs in biological imaging has been extensively studied. CDs are considered as the new internal fluorescence probe.

In this study, a solvothermal method was developed to synthesise yellow fluorescent carbon dots (Y-CDs) by using tartaric acid and urea as carbon and nitrogen sources, respectively. The Y-CDs could maintain fluorescence stability at different pHs and temperatures. Their Stokes shifts were large (4038 cm^−1^) and QY was high (15.9%). The Y-CDs could quench cobalamin quantitatively through the inner filter effect (IFE) and detect VB12 in real samples with high recycling rates. In addition, the Y-CDs were found to be applicable as fluorescent probes for MDA-MB-231 cells because the CDs could enter the nucleus smoothly.

## 2. Materials and Methods

### 2.1. Experimental Reagents and Instruments

All reagents used in the experiments are shown in Table 1. All instruments in this study are shown in Table 2.

In this manuscript, the MDA-MB-231 cells were cultivated by professor Xinguo Zhang. The place these cells were cultured was the school of pharmaceutical sciences, Southern Medical University.

### 2.2. Preparation of N-Doped Y-CDs

The one-step hydrothermal method was conducted to synthesise N-doped Y-CDs with L-tartaric acid, urea, and DMF as the raw materials [22,23,24]. First, 2.0 g of L-tartaric acid and 1.0 g of urea were dissolved in 10 mL of DMF. After ultrasonication for 5 min, the mixture was transferred to a 40 mL polytetrafluoroethylene (PTEF)-lined vessel. Next, the vessel was placed in a stainless steel hydrothermal reactor and heated at 180 °C for 4 h in an electric blast drying oven. After the reaction, 20 mL of absolute alcohol was added to dilute the solution in order to remove macromolecular impurities. Then, the mixture was subjected to centrifugation at 8000 rpm for 5 min. The supernatant was filtered using a 0.22 μm Millipore filter. After vacuum distillation, the mixture was freeze-dried for 24 h to obtain the Y-CDs.

### 2.3. Test Methods

#### 2.3.1. Scanning Electron Microscopy (SEM)

SEM was conducted to observe CD sample morphology and size. For sample preparation, the CD samples were first mixed with absolute alcohol. Upon ultrasound irradiation for 10 min, the homogeneous suspension was formed. Next, a conductive resin was stuck to a copper platform. A total of 1–2 drops homogeneous suspension were dropped on the conductive resin by a capillary, which avoided the contact between the capillary and conductive resin. After air drying, the samples were subjected to gold sputtering. The JSM-7001F system was used for SEM, and the accelerating voltage was set to 15 kV.

#### 2.3.2. Transmission Electron Microscopy (TEM)

In order to acquire the information about the internal structure and morphology of the CD samples, TEM was conducted. For sample preparation, the CD samples were first dispersed in absolute alcohol. After ultrasound irradiation for 5 min, a homogeneous suspension was formed. A few drops of the homogeneous suspension were dropped on a copper mesh by a capillary. After air drying, the JEM-2100F system was used for TEM (FEI company, Columbia, SC, USA).

#### 2.3.3. X-ray Diffraction (XRD) Analysis

The crystalline and crystalline phase purity of the samples were analysed by XRD (Shimadzu corporation, Kyoto, Japan). The Cu target was used as the radiant. The testing voltage was set to 40 kV. The testing current was set to 26 mA. The scanning range was 10°–80°. The scanning speed was 10°/min.

#### 2.3.4. Infrared (IR) Spectroscopy

IR spectroscopy was conducted to gain information about the functional groups of the CD samples. The IR spectrum of KBr pellets was considered as the blank control. Moderate KBr was weighed and mixed with the CD samples uniformly (1:200). The mixture was ground to powder. This powder was placed under an IR lamp for drying, which avoided water interference. After tablet compression, the samples were placed into an infrared spectrometer (Thermo Fisher Scientific technology company, Waltham, MA, USA). The wavenumber range was 400–4000 cm^−1^. The resolution rate was 4 cm^−1^.

### 2.4. Determination of Y-CD Fluorescence

Absolute alcohol was used as the solvent to prepare a Y-CD solution (2.5 mg/mL). Its emission spectrum was collected from 460 to 800 nm on an FLS980 fluorescence analyser in transient and steady states. The excitation wavelength was 450 nm. The slit width was 2.0 nm, and the detention time was 0.1 s. The illuminant used for emission spectroscopy and the photostability test was a xenon lamp (450 W). A 320 nm laser was used for the fluorescence lifetime test.

### 2.5. Interference Factor of Y-CDs Fluorescence

(1) Absolute alcohol, distilled water, carbinol, acetone, and PBS buffer solution (pH 7.40) were used as solvents to repair Y-CD solutions (2.5 mg/mL). From each solution, 3 mL was extracted for fluorescence test.
(2) Absolute alcohol was used as a solvent to prepare the Y-CD solution (2.5 mg/mL). The excitation wavelength was varied from 400 nm to 500 nm to obtain excitation-dependent spectra by using 3 mL of the solution.
(3) Absolute alcohol was used as a solvent to prepare the Y-CD solutions (2.5, 5, 15, 45, and 150 mg/mL). The solution (3 mL) was excited using 450 nm-wavelength light to obtain concentration-dependent spectra.
(4) NaOH solution (2 mol/L) and HCl solution (1 mol/L) were added to a PBS buffer to adjust its pH to 2–12. The PBS solutions with different pHs were used as solvents to prepare the Y-CD solutions (2.5 mg/mL). The fluorescence spectra of the Y-CDs (3 mL) at various pHs were collected.
(5) Absolute alcohol was used as a solvent to prepare the Y-CD solution (2.5 mg/mL). The excitation wavelength was 450 nm. Using 3 mL of the solution, the fluorescence emission spectra (460–800 nm) were scanned from 20 to 65 °C at 5 °C intervals. In the thermostat, the temperature was held for 5 min with every increase of 5 °C.
(6) NaCl solutions (0.4–1.2 mol/L) were used as solvents to prepare the Y-CD solutions (2.5 mg/mL). Next, 3 mL of these solutions were scanned by a fluorescence spectrometer to investigate the effect of ionic strength.

### 2.6. Detection of QYs of Y-CDs

A comparison measurement method was employed in this experiment. Rhodamine 6 g (r6g) with a *QE* of 98% was used as a standard. The emission and UV-vis spectra of r6g and Y-CDs were collected, and the QY of Y-CDs (*x*) can be calculated by Equation (1).

(1) $$QE_{x}=QE_{st}\times \frac{I_{x}}{I_{st}}\times \frac{\eta_{x}^{2}}{\eta_{xt}^{2}}\times \frac{A_{st}}{A_{x}}$$

where *I* is the integral intensity of the fluorescence emission spectrum, *η* is the solvent refractive index, and *A* is the absorbance.

### 2.7. Testing and Calculation of Fluorescence Lifetime

For the test, an FLS980 transient and steady fluorescence analyser was equipped with a 450 nm laser. The monitoring wavelength was 550 nm. The fluorescence lifetime of the Y-CDs can be calculated by Equation (2).

(2) $$I(t)=I_{0}+A_{1}\exp(-\frac{t}{\tau_{1}})+A_{2}\exp(-\frac{t}{\tau_{2}})$$

where *I*(*t*) is the fluorescence intensity of the Y-CDs at 550 nm, *I*_0_ is the initial fluorescence intensity (*t* = 0), *A* is the fluorescence lifetime constant, and *τ* is the fluorescence lifetime of the decaying exponential. The average fluorescence lifetime can be calculated by Equation (3).

(3) $$\tau^{*}=\frac{\int_{0}^{\infty }tI(t)dt}{\int_{0}^{\infty }I(t)dt}$$

### 2.8. Fluorescence-Based Cobalamin Detection

The fluorescence-based detection of cobalamin is a simple and rapid analytical process. First, 100 μL of Y-CD solution (6 mg/mL), 2100 μL of PBS solution (pH 7.400), and 800 μL of VB12 solution were added to a cuvette and mixed thoroughly. The emission spectra under 450 nm excitation were measured. For real sample analysis, cobalamin solutions of different concentrations were added to the veterinary cobalamin injection (0.5 mg/mL). The fluorescence spectra of these samples were detected as mentioned above, and the measurement was conducted in triplicate. The corresponding recycling rate and relative standard deviation (RSD) were calculated.

### 2.9. Selective Research on Cobalamin Detection

Selectivity is important in real sample analysis. In a system of cobalamin, common vitamins were used as disruptors. At the relevant concentrations of disruptors, the fluorescence spectra were collected. According to the effects of the disruptors on the fluorescence of the Y-CDs, the selectivity of Y-CDs for cobalamin detection was verified [25,26,27,28].

### 2.10. Study on the Mechanism of Cobalamin Detection

#### 2.10.1. Effect of Cobalamin on Y-CD UV Spectrum

A solution of the Y-CDs (50 μg/mL) and a cobalamin solution (200 μM) were mixed, and the UV absorption spectrum of the mixture was scanned from 400 to 750 nm.

#### 2.10.2. Effect of Cobalamin on Y-CD Fluorescence Lifetime

A solution of the Y-CDs (50 μg/mL) and a cobalamin solution (0–200 μM) were mixed. The experimental conditions were the same as those mentioned in Section 2.7. At different cobalamin concentrations, the fluorescence lifetimes of the Y-CDs were tested.

#### 2.10.3. Cytotoxicity Investigation and In Vitro Cell Imaging

MDA-MB-231 human breast cancer cells were cultured in RMPI-1640 media containing 10% Australian foetal bovine serum and 1% penicillin–streptomycin solution. First, the MDA-MB-231 cells were inoculated to a 96-well plate and placed into a 5% CO_2_ incubator at 37 °C for 24 h. The culture solution is H_2_O. When the cell density reached about 5 × 10^3^–10 × 10^3^ per well, the culture medium in the well was suctioned and the cells were washed with PBS solutions (pH 7.40). Next, 100 μL of the culture medium containing 2, 8, 15, 20, 30, and 150 mg/mL Y-CDs was added. Two groups of the samples were prepared following the abovementioned steps. These samples were placed in a 5% CO_2_ incubator at 37 °C for 24 h. The culture solution is H_2_O. After incubation, the Y-CD solution was extracted, and the cells were washed with PBS (pH 7.40). Next, 10 μL of CCK8 solution was added into each well and incubated for 4 h. After incubation, the absorption value at 450 nm was determined using a microplate reader.

MDA-MB-231 cells (1 mL) were added into a 15 mm glass bottom cell culture dish for inoculation, and placed in a 5% CO_2_ incubator at 37 °C for 12 h. Next, the culture medium was extracted and replaced by 1 mL of the culture medium containing 20 mg/mL of Y-CDs. After incubating for 1 h and 4 h, the medium was discarded and replaced with 2 mL of 4% paraformaldehyde (POM). The solution was stewed for 20 min to fix the cells. Then, a PBS solution (pH 7.40) was used to wash POM. Finally, an anti-fluorescence quenching mounting medium was added, and the cells were observed under an inverted LSCM.

## 3. Results

### 3.1. Structure and Morphology Characterisation of Y-CDs

**Figure 1:**
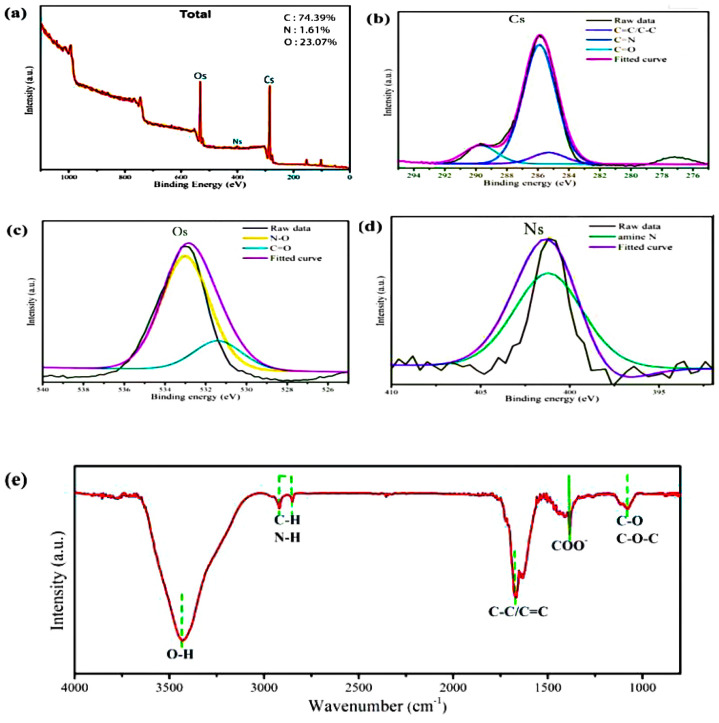
(**a**) XPS survey scan of the Y-CDs. High-resolution XP spectra of (**b**) Cs, (**c**) Os, and (**d**) Ns for the Y-CDs. (**e**) FT-IR spectra of the Y-CDs.

The elemental composition, functional groups, and morphological structure of the Y-CDs were analysed by XPS, FT-IR spectroscopy, XRD, and TEM. Figure 1a shows the full XP spectrum, wherein three binding energy peaks (Cs, Os, and Ns) are detected. The elemental proportions of C, O, and N are 74.39%, 23.07%, and 1.61%, respectively. Figure 1b shows the peak fitting results of Cs, and the peaks at 284.3 eV, 284.8 eV, and 288.6 eV correspond to C-C/C=C, C=N, and C=O, respectively. Similarly, N-O, C=O, and amino groups are identified in the high-resolution XP spectra of Os and Ns [29,30,31]. These results agree with the FT-IR spectrum in Figure 1e. The strong absorption peak at 3397 cm^−1^ is related to O-H stretching vibration. The C-H or N-H functional groups show absorption peaks between 2760 cm^−1^ and 2790 cm^−1^. The peaks at 1668 cm^−1^ and 1063 cm^−1^ are assigned to the C=C and C-O-C functional groups, respectively. These functional groups are responsible for the high water solubility and stability of the Y-CDs.

**Figure 2:**
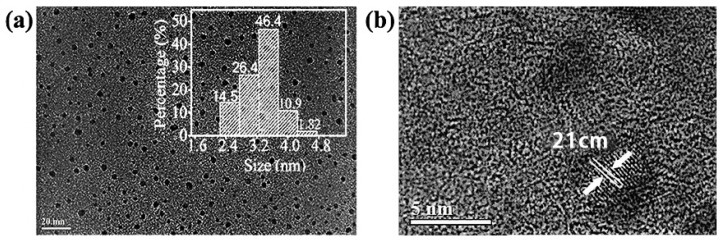
TEM images of the Y-CDs. (**a**) TEM images with 1nm resolution ration, (**b**) TEM images with 0.1nm resolution ration.

Figure 2a,b show the TEM images of the Y-CDs at different magnifications. As shown in Figure 2a, the small particles in the Y-CDs disperse evenly. The interpolated image is the grain diameter distribution map. The distribution of particles sizes is between 2 nm and 5 nm, and is largely concentrated at 3.8 nm. From Figure 2b, with the geometric measurement in MATLAB, the cell interval was found to be 0.21 nm, which is similar to that in the graphene layers.

### 3.2. Fluorescence Stability of Y-CDs

**Figure 3:**
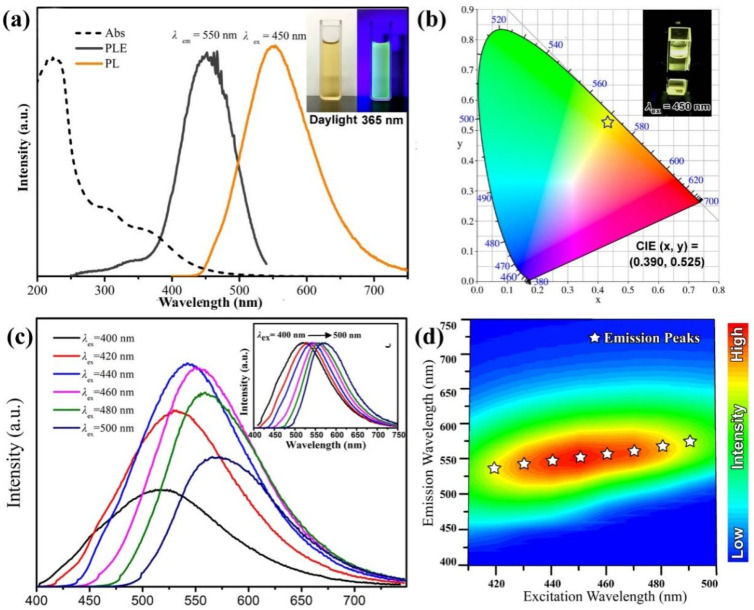
(**a**) Absorption, excitation, and emission spectra, and (**b**) CIE coordinates of the Y-CDs (The Pentagram). (**c**) PL spectra of the Y-CDs excited at different wavelengths. (**d**) Excitation-emission matrix of the Y-CD solution.

From the interpolated map in Figure 3a, the alcohol solution with 2.5 mg/mL Y-CDs was found to be a flaxen, transparent, and clear liquid under natural light. Under 395 nm UV radiation, the solution emits yellowish green fluorescence. From Figure 3b, under 450 nm UV radiation, the solution emits yellow fluorescence. From the UV absorption curve in Figure 3a, the UV absorption area exists at <300 nm. It is caused by the π−π* transition induced by C=C and C=N. The absorption band exists at 350–500 nm. It is caused by the n-π* and π-π* transitions induced by C=O and the aromatic group structure. The band gap transition at 450 nm is caused by the interface excitation of the Y-CDs. From Figure 3a, the optimum excitation wavelength and maximum emission wavelength of the Y-CD alcohol solution are 450 nm and 550 nm, respectively. The chromatic coordinates were as follows: CIE (x, y) = (0.390, 00.525). From Figure 3b, the solution was found to emit yellow fluorescence. According to the test and calculation, the quantum efficiency of the Y-CD alcohol solution and aqueous solution were found to be 16.7% and 4.5%, respectively. In the view of chemistry, the hydrogen bond in alcohol is stronger than that in water. Therefore, the molecular dipole moment affects the surface electronic state further, which increases the quantum efficiency of the Y-CD alcohol solution. In this study, all luminescent property tests of the Y-CDs were carried out in the alcohol solution. There are many luminescence centres in the CDs. Thus, the PL characteristics related to excitation were considered as special characteristics. When the excitation wavelength increases from 400 nm to 500 nm, the emission peaks gradually shift from 510 nm to 575 nm. The emission characteristics of the Y-CDs is related to the excitation wavelength. This may be related to the differences in the luminescence centres or the radiation contribution of the surface states. From Figure 3c, with the change in the excitation wavelength, the emission wavelength of the Y-CD alcohol solution also red shifts and the fluorescence intensity decreases, which is attributed to the Y-CDs possessing multiple luminescence centres. From Figure 3d, when the excitation wavelength is 450 nm, the Y-CD alcohol solution emits fluorescence at 550 nm. The fluorescence intensity reaches the maximum.

### 3.3. Fluorescence Stability of Y-CDs

**Figure 4:**
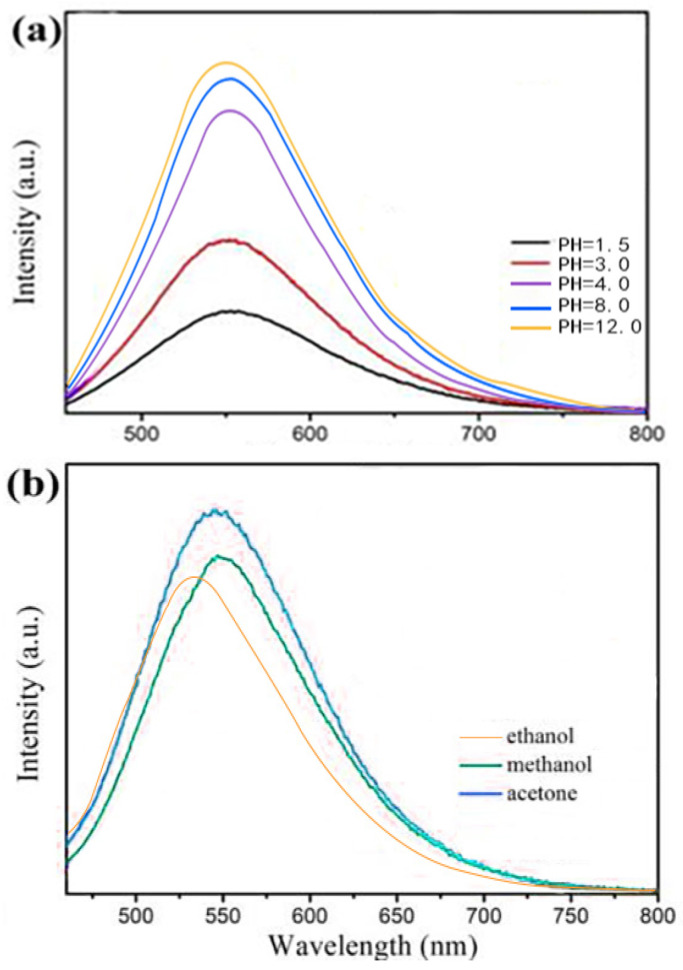
PL spectra of the Y-CDs (**a**) at different pHs (*λ_ex_* = 450 nm) and (**b**) in different solvents (*λ_ex_* = 450 nm).

Fluorescence stability is an important property governing the application scope of CDs. Figure 4a shows the pH-dependent fluorescence spectra of the Y-CDs. The emission wavelength does not change significantly. In the pH range of 1.5–4, the fluorescence intensity increases rapidly because the functional groups on the Y-CD surface are hydrolysed under the strong acid condition. When the pH value is 4–8, the fluorescence intensity increases and remains stable. The fluorescence intensity is slightly enhanced under an alkaline condition (pH 8–12). Different solvents were used to dissolve the Y-CDs at the same concentration. As shown in Figure 4b, the fluorescence intensity of the Y-CDs in ethanol is higher than those in carbinol, acetone, distilled water, and the buffer solution. Different solvent polarities cause the rearrangement of the surface electrons of the Y-CDs. The dipole moment changes greatly and the fluorescence intensity of the Y-CDs decreases.

**Figure 5:**
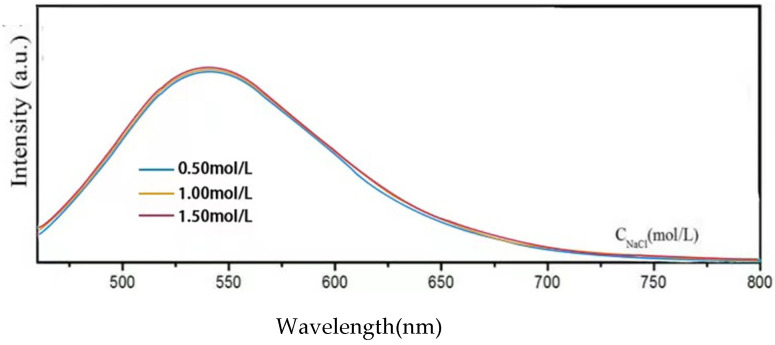
PL spectra of the Y-CDs in NaCl solutions with different ionic strengths (*λ_ex_* = 450 nm).

In Figure 5, the fluorescence intensity of the Y-CDs (2.5 mg/mL) remains unchanged in 0.50–1.50 M NaCl solutions, confirming that the Y-CDs exhibit good salt tolerance at high ionic strength. Therefore, the Y-CDs demonstrate excellent fluorescence stability at different pHs, temperatures, and ionic strengths.

### 3.4. Fluorescence-Based Detection of Cobalamin

**Figure 6:**
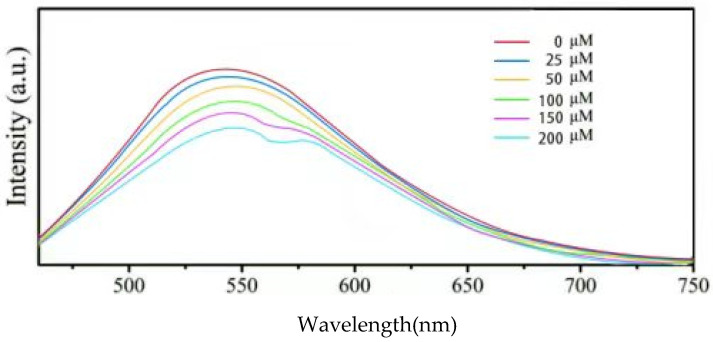
Fluorescence responses of the Y-CDs in the presence of VB12 at different concentrations (*λ_ex_* = 450 nm, *λ_em_* = 550 nm). The inset shows the corresponding photographic images of Y-CDs with 0 μM and 200 μM VB12 under 395 nm UV illumination.

Considering their excellent stability, the Y-CDs were used for VB12 detection. As shown in Figure 6, the emission intensity at 530 nm quenches in the presence of VB12 at different concentrations (0, 25, 50, 100, 150, 200 μM). With the increasing VB12 concentration, the emission intensity of the Y-CDs decreases gradually. When the concentration is higher than 100 μM, the emission peak is divided into a doublet because of the spectral overlap of the VB12 absorption peak and the Y-CD emission peak. After gaussian decomposition, as shown in Figure 6, the Y-CD emission peak is divided into two peaks (542 nm and 600 nm), suggesting the presence of at least two luminescence centres in the Y-CDs. The absorption peak of VB12 overlaps with the peak at 542 nm. Therefore, the IFE occurs between the VB12 absorption peak and the 544 nm absorption peak, dividing the Y-CD emission peak gradually.

**Figure 7:**
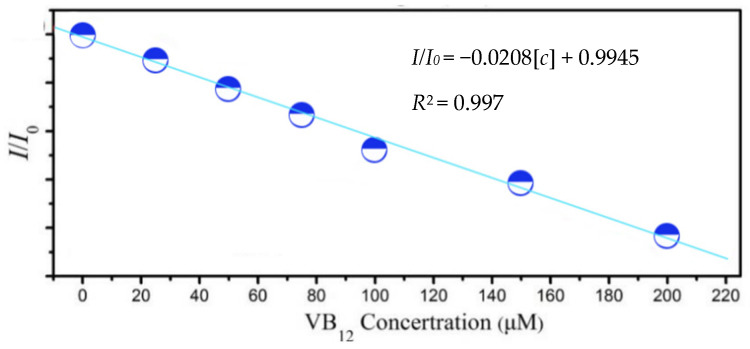
Relationship between the emission integrated area and VB12 concentration.

As shown in Figure 7, the fluorescence intensity ratio at 530 nm (*I/I*_0_) shows a linear relationship with the cobalamin concentration. The Stern–Volmer equation was used: *I/I*_0_ = −0.0208[*c*] + 0.9945, where *I*_0_ is the fluorescence intensity of the Y-CDs before adding VB12, *I* is the fluorescence intensity of the Y-CDs after adding VB12 at different concentrations, and [*c*] is the concentration of VB12. The correlation coefficient (*R*^2^) is 0.997, and the limit of detection (LOD) is 2.101 μmol/L.

### 3.5. Quenching Mechanism for Cobalamin Detection

**Figure 8:**
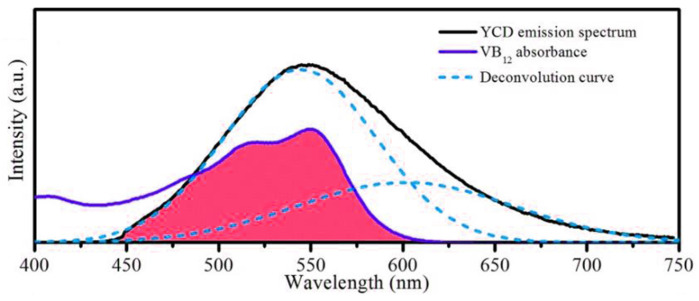
Overlap of the VB12 absorption spectrum and Y-CD emission spectrum (The red region is the repeat region).

The systematic fluorescence quenching mechanism includes static quenching, dynamic quenching, Forster resonance energy transfer (FRET), photo-induced electron transfer (PET), and IFE. Different quenching mechanisms occur under different conditions. The fluorescence emission of the Y-CDs and the UV absorption spectra of VB12 are shown in Figure 8. A large overlapping area is found at 430–620 nm, suggesting that VB12 can absorb a part of the fluorescence emitted by the Y-CDs. When the Y-CDs constantly emit fluorescence, the quantity of absorption fluorescence depends on VB12 concentration. Therefore, VB12 can quench the fluorescence of the Y-CDs, showing a linear relationship within a specific concentration range. According to the nature of spectral overlap, it can be deduced that the quenching mechanism may be related to the FRET or IFE. For verification of the quenching mechanism, fluorescence lifetime attenuation must be considered.

**Figure 9:**
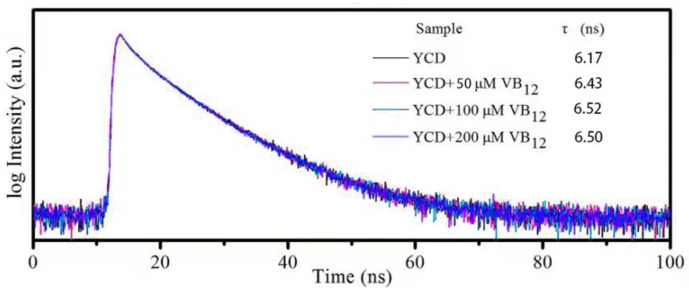
Time-resolved decay of the Y-CDs in the presence and absence of VB12. The inset table presents the lifetime value.

Figure 9 shows the time-resolved decay of Y-CDs in the presence and absence of VB12. When the VB12 concentration varies from 0 to 200 μM, the fluorescence lifetime does not change. This indicates that no new chemical compound is generated during the quenching process, which is contrary to the FRET mechanism. When the fluorophore coexists with the other absorbing materials, the stimulated luminescence or emission light is absorbed by the fluorophore or other absorbing materials, quenching the fluorescence. This phenomenon is the fluorescence IFE. In this process, no new chemical compound is generated, and the quenching mechanism of the Y-CDs is more likely to be the IFE.

### 3.6. Content Assay Via Cobalamin Injection

To explore the practical application of the Y-CDs, veterinary cobalamin injection is applied for content assay. The concentration of cobalamin injection (0.5 mg/mL) is 368.903 µM, exceeding the detection range of the Y-CDs. Therefore, the cobalamin injection is diluted to 92.226 µM. The cobalamin concentration is detected as 92.283 µM. The absolute error value is 0.057 µM. The relative error value is 0.06%. To compare the preliminary fluorescence detection, the organic dyestuff and quantum dots are used as the fluorescent carbon dots. Under the same experimental environment, the cobalamin content assay is carried out. Through comparison and analysis, the error in the Y-CD fluorescence detection is the minimum. Based on the results summarised in Table 3, this method is more suitable for actual cobalamin detection.

### 3.7. Cytotoxicity Test and In Vitro Cell Imaging of Y-CDs

**Figure 10:**
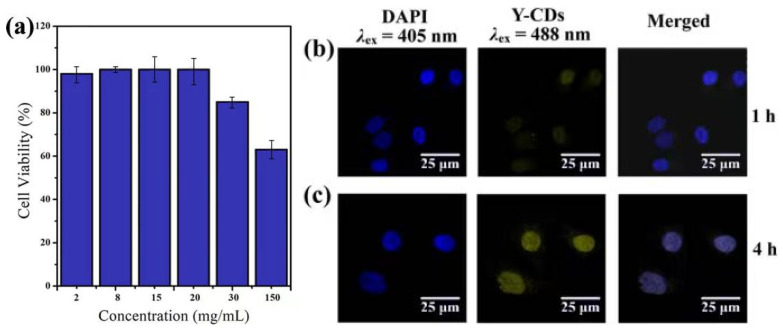
(**a**) Cytotoxicity effect of the Y-CDs on MDA-MB-231 pancreatic cancer cells. (**b**,**c**) Microscope images of MDA-MB-231 cells incubated with 20 mg/mL Y-CDs for 1 h and 4 h, respectively. The left panels are bright field images of the cancer cells, the middle panels are the fluorescence images, and the last panel is the merged image of the bright field and fluorescent images.

Before the cell imaging experiment, the cytotoxicity of the Y-CDs to MDA-MB-231 cells was evaluated using the CCK-8 method. The cell survival rate shown in Figure 10a was determined after the cells were co-cultured with different concentrations of the Y-CDs for 4 h. When the Y-CD concentration reaches 20 mg/mL, the cell survival rate is higher than 90%. When the concentration is higher than 20 mg/mL, the cell survival rate decreases gradually. Combining the effect of the cell survival rate and Y-CD concentration on the fluorescence intensity, 20 mg/mL of Y-CDs was chosen for the cell imaging experiment.

The blue fluorescence emitted from a DAPI dye under 441 nm laser excitation is shown on the left in Figure 10b,c. The cell locations can be marked because the DAPI dye enters the nucleus. After the Y-CDs and cells are incubated for 1 h, a few Y-CDs entered the nucleus. Under the excitation of a 490 nm laser, the Y-CDs exhibit faint yellow fluorescence in the nucleus. After incubation for 4 h, the yellow fluorescence enhances, and the cell contour can be observed, indicating that the Y-CDs entered the nucleus after 4 h. These observations confirm that the Y-CDs have good biocompatibility and can be used in cell imaging.

## 4. Conclusions

In this study, tartaric acid and urea were used as raw materials to prepare Y-CDs via a green and simple solvothermal method. The mean size of Y-CDs was 6 nm, and the size distribution was narrow. The Y-CDs exhibited strong excitation-dependent fluorescence at 530 nm with a high QY of 15.9%. The excellent fluorescence stability of the Y-CDs was verified at different pHs, temperatures, and ionic strengths. The Y-CDs could detect cobalamin sensitively and selectively through the IFE mechanism. The emission intensity of the Y-CDs and cobalamin concentration showed a linear correlation at 0–200 μM. The related coefficient was 0.997, and the detection limit was 2.101 μmol/L. The feasibility of this quenching method was demonstrated by detecting cobalamin in real samples. Moreover, the MDA-MB-231 cells co-cultured with the Y-CDs showed insignificant cytotoxicity and exhibited yellow fluorescence under 490 nm excitation. The result indicated that the Y-CDs possess imaging depth because they are small enough to enter the nucleus. Therefore, the Y-CDs can be applied in cobalamin detection and as a multifunctional fluorescent probe for cell imaging.

## Figures and Tables

**Table 1:** Chemical reagents used in the experiments.

Reagent	Purity	Manufacturer	Purpose
L-tartaric acid	Analytical pure	Aladdin Reagent Co., Ltd., Shanghai, China	Carbon source of CD
Urea	Analytical pure	GHTECH Co., Ltd., Guangzhou, China	Nitrogen source of CD
N-Dimethylformamide (N-DMF)		Aladdin Reagent Co., Ltd., Shanghai, China	Nitrogen source of CD
Acetone	Analytical pure	DaMao chemical reagent factory, Tianjin, China	Solvent for CD
Carbinol	Analytical pure	DaMao chemical reagent factory, Tianjin, China	Solvent for CD
Absolute alcohol	Analytical pure	DaMao chemical reagent factory, Tianjin, China	Solvent for CD
Concentrated hydrochloric acid	Analytical pure	GHTECH Co., Ltd., Guangzhou, China	PH tuning agents
Sodium hydroxide	Analytical pure		Salt content test
Sodium chloride	Analytical pure	Aladdin Reagent Co., Ltd., Shanghai, China	Salt content test
Phosphate buffer (pH 7.4)	Analytical pure	Yuanye Bio-Technology Co., Ltd., Shanghai, China	Buffer solution
Standards for cobalamin	Analytical pure	Aladdin Reagent Co., Ltd., Shanghai, China	Cobalemin source
Veterinary cobalamin injection			Cobalemin source
DEME culture medium	Analytical pure	Tianjun Biotechnology Co., Ltd., Guangzhou, China	Cellular toxicity and imaging agents
4% polyformaldehyde stationary liquid	Analytical pure	Bairui Biotechnology Co., Ltd., Shanghai, China	Cellular toxicity and imaging agents
Gibco Research Grade EU Approval Serum	Analytical pure	Gibco Bio-Technology Co., Ltd., New York, NY, USA	Cellular toxicity and imaging agents
RPMI-1640 culture medium	Analytical pure	Gaochuang Technology Instrument Co., Ltd., Shanghai, China	Cellular toxicity and imaging agents
Penicillin–streptomycin mixture	Analytical pure	Yuanye Bio-Technology Co., Ltd., Shanghai, China	Cellular toxicity and imaging agents
Anti-fluorescence quenching mounting medium	Analytical pure	Cida Bio-Technology Co., Ltd., Guangzhou, China	Fluorescent imaging agent

**Table 2:** Experimental instruments.

Instruments	Model	Manufacturer	Purpose
Steady/transient state X-ray fluorescence (XRF) spectrometer	FLS980	Edinburgh Instruments company, Edinburgh, Britain	Luminescent property
Fourier-transform infrared (FT-IR) spectroscope	6700 FT-IR	Thermo Fisher Scientific technology company, Waltham, MA, USA	Surface group
UV-visible-near infrared light spectrophotometer	UV-3600	Shimadzu corporation, Kyoto, Japan	Absorption property
Transmission electron microscope	FEI TECNAI G2 F20	FEI company, Columbia, SC, USA	Particle morphology
X-ray photoelectron (XP) spectroscope	AXIS ULTRA DLD	Shimadzu corporation, Kyoto, Japan	Particle composition
Liquid nitrogen cryostat	OptistatDN-V2	Oxford instrument technology Co., Ltd., Shanghai, China	Temperature control
Elispot analyser	CTL S6 Versa	Thermo Fisher Scientific technology company, Waltham, MA, USA	Cellular imaging
Constant magnetic stirring	85-2	Aohua instrument Co., Ltd., Changzhou, China	CD synthesis
Table-top high-speed centrifuge	TG16-WS	Xiangyi laboratory Instrument Development Co., Ltd., Xiangtan, China	CD purification
Collector type constant-temperature heating magnetic stirrer	DF-101S	Yuhua instrument Co., Ltd., Gongyi, China	CD synthesis
Electronic analytical balance	AX124 ZH/E	OHAUS instrument Co., Ltd., Newark, DE, USA	Sample weighing
Camera obscura UV analyser	ZF-20D	Yuhua instrument Co., Ltd., Gongyi, China	CD emission observation
Electric blast drying oven	DHG-9145A	Yiheng scientific instrument Co., Ltd., Shanghai, China	Cellular imaging
Laser confocal microscope (LSCM)	LSM 880 with Airyscan	Carl Zeiss AG Co., Ltd., Oberkochen, Germany	Cellular imaging
Freeze dryer	Freezone6L	Labconco instrument and equipment Co., Ltd., Butler, PA, USA	CD purification

**Table 3:** Different methods for determination of animal vitamin B12 in real samples; the “found” value is the value of three parallel experiments.

Fluorescent Carbon Dots	Found (µM)	Absolute Error Value (µM)	Relative Error Value (%)
Y-CDs	92.283	0.057	0.06
Dyestuff	90.574	1.652	1.80
Quantum dots	90.266	1.960	2.20

## Data Availability

Not applicable.
